# Atypical Chronic Myelogenous Leukemia, *BCR-ABL1* Negative: Diagnostic Criteria and Treatment Approaches

**DOI:** 10.3389/fonc.2021.722507

**Published:** 2021-11-17

**Authors:** Panagiotis T. Diamantopoulos, Nora-Athina Viniou

**Affiliations:** First Department of Internal Medicine, Laikon General Hospital, National and Kapodistrian University of Athens, Athens, Greece

**Keywords:** atypical chronic myelogenous leukemia, myelodysplastic syndrome/myeloproliferative neoplasm, diagnostic criteria, treatment, molecular characterization

## Abstract

Atypical chronic myelogenous leukemia (aCML), *BCR/ABL1* negative is a rare myelodysplastic/myeloproliferative neoplasm, usually manifested with hyperleukocytosis without monocytosis or basophilia, organomegaly, and marked dysgranulopoiesis. In this review, we will discuss the classification and diagnostic criteria of aCML, as these have been formulated during the past 30 years, with a focus on the recent advances in the molecular characterization of the disease. Although this entity does not have a definitive molecular profile, its molecular characterization has contributed to a better understanding and more accurate classification and diagnosis of aCML. At the same time, it has facilitated the identification of adverse prognostic factors and the stratification of patients according to their risk for leukemic transformation. What is more, the molecular characterization of the disease has expanded our therapeutic choices, thoroughly presented and analyzed in this review article.

## Introduction

Atypical chronic myelogenous leukemia (aCML), *BCR/ABL1* negative is a rare disorder classified into the category of myelodysplastic/myeloproliferative neoplasms (MDS/MPN), according to the 2016 revision of the World Health organization (WHO) classification of myeloid neoplasms and acute leukemia (1). It is, by definition, a *BCR-ABL1*-negative clonal disorder sharing myelodysplastic and myeloproliferative features. Due to its rarity, the diagnostic criteria for aCML have been changing since its first description while there are no established standards of care for patients with this condition; hence we will focus on the diagnostic criteria of the disease, new molecular characteristics that have emerged during the last few years, some of which have already been incorporated in the latest diagnostic criteria of the WHO, the proposed risk stratification systems, and the available treatment approaches for aCML.

## Epidemiology, Clinical and Laboratory Characteristics

The incidence of aCML is low, a fact leading to limited knowledge about this disease entity. Its true incidence is largely unknown, since it is usually estimated in comparison to that of (*BCR-ABL1*-positive) CML at about one to two cases per 100 cases of CML (2). The cases reported so far concern adults. Patients usually present in the seventh or eighth decade of life and there is an apparent male predominance (1:1 to 4:1 in several case series).

The patients usually present with organomegaly and hyperleukocytosis. Splenomegaly has been reported in 50% to 75% of the patients while in one patient series, hepatomegaly has been reported to be present in 49% of the patients. The median white blood cell (WBC) count at presentation has been reported to fluctuate between 23x10^9^/L to 97x10^9^/L in several case series. The patients are usually moderately to severely anemic [median hemoglobin (Hb) level, 8.6 g/dL to 11.7 g/dL], while about two thirds of them are transfusion dependent. The platelet count may be decreased or normal but can also be found increased in several cases (3). Monocyte and basophile counts are usually within normal range while immature myeloid precursors (promyelocytes, myelocytes, metamyelocytes) are usually present in the peripheral blood smear (10% to 20%), but peripheral blasts are generally absent or low. The neutrophils are highly dysplastic in most cases with typical pseudo-Pelger-Huet changes and hypogranularity of the cytoplasm while dyserythropoiesis and dysmegakaryopoiesis may also be present (3). The bone marrow is hypercellular, with an increased myeloid-to-erythroid ratio (usually up to 10:1) while marrow fibrosis may be present. By definition, blood or bone marrow blasts are less than 20%..

## Classification and Diagnostic Criteria

In the late 80s, the first reports of the disease came to light, with the scientific community wondering whether a Philadelphia-negative CML exists (4). This lead to the identification of “masked” or “hidden” cases of *BCR/ABL1* translocation (5–8), and to the understanding that “CML-like” changes can be found without the diagnostic hallmark of CML. At the same time, cases with features compatible with chronic neutrophilic leukemia (CNL) (9) or chronic myelomonocytic leukemia (10, 11) but atypical presentation were identified, leading to the initial descriptions of the clinical and laboratory characteristics of the disease that eventually led to its classification in the group of chronic myeloid leukemias (12). The French-American-British (FAB) Cooperative Leukaemia Group also classified the disease in 1994 as a subtype of chronic myeloid leukemia with distinct features such as the low basophil and monocyte count, granulocytic dysplasia, presence of immature granulocytes up to 10%-20% of WBC, blasts <2%, and the absence of Philadelphia (Ph) chromosome and the *BCR-ABL1* fusion gene (13). The WHO 2001 classification had minimal differences from the original FAB classification, mainly referring to the marked multilineage dysplasia and the blast percentage in the bone marrow at <20%. In 2008, the new WHO classification added the threshold of 13x10^9^/L to define leukocytosis while it also proposed the limit of 10% of total WBC for monocytes. Moreover, it limited dysplasia to dysgranulopoiesis and for the first time it required the absence of other rearrangements besides that of *BCR-ABL1* (2). Finally, the latest WHO 2016 classification (1) emphasized molecular changes in newly described genes (*ETNK1*, *SETBP1*) and the absence of rearrangement of PDGFRA, PDGFRB, FGFR1, and PCM1-JAK2. Moreover, for the first time, it was added as an exclusion criterion that WHO criteria for other myeloproliferative neoplasms should not be met. Finally, it is the first time that marrow cellularity is commented on. Nevertheless, there is no quantification of bone marrow cellularity and dysplasia in any of the available diagnostic criteria as is usually the case in other disease entities. Although a major feature of aCML, prominent dysgranulopoiesis is not further specified qualitatively and, especially, quantitatively. Granulocytic dysplasia includes pseudo-Pelger-Huët cells, other types of abnormal segmentation of the nucleus or abnormal chromatin clumping, and reduced cytoplasmic granularity. Although, dysgranulopoiesis is the main morphologic feature of the disorder, dyserythropoiesis and, especially, dysmegakaryopoiesis are not rare, although more subtle. These defining morphologic features help distinguish aCML from other entities such as CML and CNL where dysplastic features are minimal or absent (14). A comprehensive list of the diagnostic criteria for aCML as these were shaped during the last 35 years is presented in **Table 1**.

**Table 1:** Diagnostic criteria of atypical chronic myeloid leukemia, *BCR/ABL1* negative.

Feature	FAB (1994)	WHO 2001	WHO 2008	WHO 2016
Ph chromosome	Absent	Absent	Absent	Absent
*BCR/ABL1*	Absent	Absent	Absent	Absent
Leukocytosis	Present	Persistent	Persistent (≥13x10^9^/L)	Persistent (≥13x10^9^/L)
Basophil count	<2%	<2%	<2%	<2%
Monocyte count	≥3% and <10%	<1x10^9^/L	<1x10^9^/L and <10% of leukocytes	<10% of leukocytes
Multilineage dysplasia	NA	Marked	NA	Dyserythropoiesis and dysmegakaryopoiesis may be present
Dysgranulopoiesis	++	NA	Marked	Present
Immature granulocytes	10-20%	>10%	NA	≥ 10% leukocytes
Blasts	>2%	<20% (bone marrow)	NA	<20% (blood and bone marrow)
Bone marrow cellularity	NA	NA	NA	Hypercellular bone marrow
Other molecular characteristics	NA	NA	No rearrangements of *PDGFRA*, *PDGFRB*, and *FGFR1*	No rearrangements of *PDGFRA*, *PDGFRB*, *FGFR1*, and *PCM1-JAK2*
				Emphasis on molecular changes (*ETNK1*, *SETBP1*)
Other factors	NA	NA	NA	WHO criteria for other MPNs not met

FAB, French-American-British; WHO, World Health Organization; Ph, Philadelphia; MPN, myeloproliferative neoplasm; NA, not available; MPN, myeloproliferative neoplasm.

In conclusion, the defining characteristics of aCML are granulocytic proliferation with marked dysgranulopoiesis, along with minimal or absent monocytosis and absence of basophilia, without *BCR/ABL1* translocation or rearrangements of genes that define other hematologic neoplasms. Because of the rarity of aCML, it remains virtually an exclusion diagnosis that should be made when other MDS and MPN can be safely ruled out. In recent years, the molecular profile of the disease is being increasingly outlined through the accumulation of data on the detection of recurrent molecular abnormalities that may contribute to the differential diagnosis of aCML. These molecular changes may be incorporated in the diagnostic criteria for the disease in the future.

The cytogenetic abnormalities in aCML, apart from the lack of the Philadelphia chromosome, are not disease-specific and include trisomy 8, deletion Y, deletion 20q, isochromosome 17(q), and other cytogenetic changes along with complex karyotype that are usually found in MDS. Nevertheless, about 50% to 80% of the cases have normal karyotypes (15, 16).

**Figure 1:**
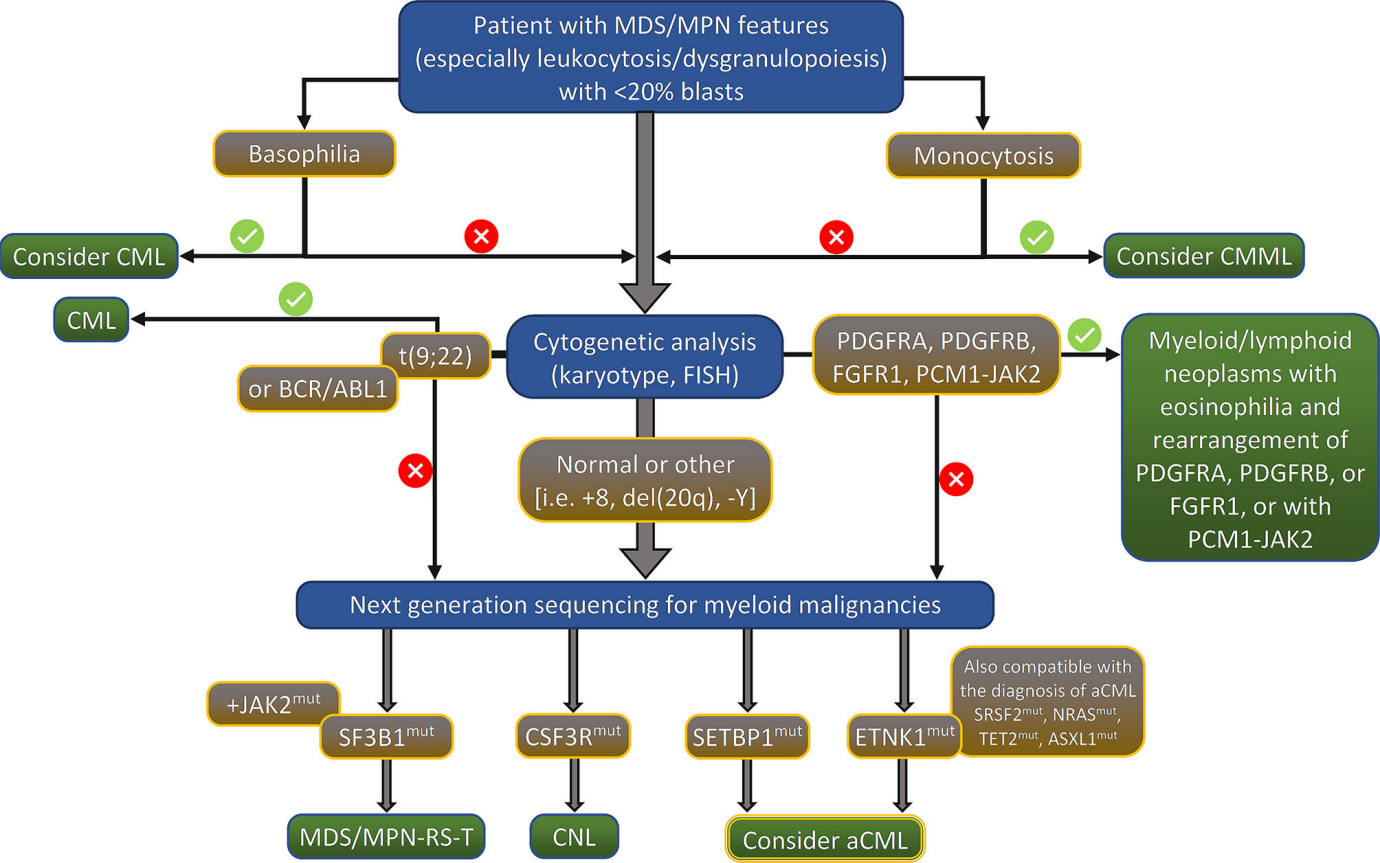
Proposed algorithm for the diagnosis of aCML. MDS/MPN, myelodysplastic syndrome/myeloproliferative neoplasm; CML, chronic myeloid leukemia; CMML, chronic myelomonocytic leukemia; FISH, fluorescent *in situ* hybridization; RS, ring sideroblasts; T, thrombocytosis; CNL, chronic neutrophilic leukemia; aCML, atypical chronic myelogenous leukemia, BCR/ABL1 negative.

Although the presence of several molecular changes can help to rule out the diagnosis of aCML, there is no definitive molecular profile of the disease. During the last few years, recurrent mutations have been increasingly identified in patients with aCML, but the percentage of patients bearing those mutations vary significantly among different studies. Frequently mutated genes (i.e. >20% of cases) are *SETBP1*, *ASXL1*, *NRAS/KRAS*, *SRSF2*, and *TET2* while a variety of genes, including *CSF3R*, *CBL*, *EZH2*, *ETNK1*, *U2AF1* and others (14, 15, 17–22) are found mutated in a lower frequency (<10% of cases). The genes found recurrently mutated in aCML, along with their chromosomal location, normal function, affected exons, mutation types, and clinical implications are listed in **Table 2** (23). Moreover, an algorithm for the diagnosis of aCML incorporating molecular data has been proposed and can be found in **Figure 1**. Since some of the mutated genes found in patients with aCML may be targetable, below we will discuss frequently mutated genes that may affect treatment decision. A list of actionable and non-actionable mutations along with potential targeted therapies can be found in **Table 3**.

**Table 2:** Recurrently mutated genes in aCML.

Gene	Chromosomal location	Normal function	Mutations	Clinical implications
*SETBP1*	18q12.3	It encodes a protein containing a ski homology region, a SET-binding region, and 3 nuclear localization signals. The protein binds to the SET nuclear oncogene which is involved in DNA replication. The SETBP1 protein is thought to control genes involved in developmental processes (e.g. nerve cell migration in the brain during fetal development).	Exon count: 15	Severe anemia and thrombocytopenia
			Nonsense substitution A>C	
			**Missense substitution A>G**	Poor prognosis
			**Synonymous substitution A>T**	
			Inframe deletion C>G	
			Frameshift deletion G>A	
			Inframe insertion C>A	
			Frameshift insertion C>T	
			Complex mutation G>C	
			c.2602G>A, p.Asp868Asn (sAML, MDS, CMML1/2, CML-BP) c.2608G>A, p.Gly870Ser (sAML, MDS, CMML1/2, CML-BP) c.2640C>A, p.Asp880Glu (sAML) c.2638G>A, p.Asp880Asn (CMML1) c.2612T>C, p.Ile871Thr (sAML, pAML,	
*ETNK1*	12p12.1-p11.2	It catalyzes the first step of the *de novo* phosphatidylethanolamine biosynthesis pathway, responsible for the phosphorylation of ethanolamine to phosphoethanolamine.	Exon count: 13	Association with altered sensitivity to the AKT kinase inhibitor capivasertib
			Nonsense substitution A>C	
			**Missense substitution A>G**	
			Synonymous substitution A>T	
			Inframe deletion C>G	
			Frameshift deletion G>A	
*CSF3R*	1p34.3	Essential for granulocytic maturation.	Exon Count: 19	Transformation to AML
		Plays a crucial role in the proliferation, differentiation, and survival of cells of the neutrophilic lineage.	Nonsense substitution A>C	Congenital neutropenia
			**Missense substitution A>G**	
			Synonymous substitution A>T	
		May function in some adhesion or recognition events at the cell surface.	Inframe deletion C>G	
			Frameshift deletion G>A	
			Inframe insertion C>A	
			Frameshift insertion C>T	
			Complex mutation G>C	
*SRSF2*	17q25.1	Necessary for pre-mRNA splicing.	Exon count: 5	Poor prognosis in MDS
		Required for formation of the earliest ATP-dependent splicing complex and interacts with spliceosome components bound to both the 5’- and 3’-splice sites during spliceosome assembly.	Nonsense substitution A>C	No prognosis impact in CMML
			**Missense substitution A>G**	
			Synonymous substitution A>T	
			Inframe deletion C>G	
			Frameshift deletion G>A	
			Inframe insertion C>A	
			Frameshift insertion C>T	
			Complex mutation G>C	
*NRAS*	1p13.2	Oncogene encoding a membrane protein that shuttles between the Golgi apparatus and the plasma membrane.	Exon count: 7	Associated with altered sensitivity to selumetinib, cediranib, ibrutinib, and dasatinib.
			Nonsense substitution A>C	
			**Missense substitution A>G**	
		The protein has intrinsic GTPase activity; it is activated by a guanine nucleotide-exchange factor and inactivated by a GTPase activating protein	Synonymous substitution A>T	
			Inframe deletion C>G	Mutations which change amino-acids 12, 13 or 61 enhance the potential of Ras to transform cultured cells and are implicated in a variety of human tumors.
			Frameshift deletion G>A	
			Inframe insertion C>A	
			Frameshift insertion C>T	
			Complex mutation G>C	
*ASXL1*	20q11.21	It encodes a chromatin-binding protein, member of the Polycomb group of proteins involved in transcriptional regulation mediated by ligand-bound nuclear hormone receptors, such as retinoic acid receptors (RARs) and peroxisome proliferator-activated receptor gamma (PPARG).	Exon count: 18	Mutations in *ASXL1* are associated with altered sensitivity to 46 drugs among them olaparib, venetoclax, and pevonedistat
			**Nonsense substitution A>C**	
			**Missense substitution A>G**	
			Synonymous substitution A>T	
			Inframe deletion C>G	
			**Frameshift deletion G>A**	
		It is thought to disrupt chromatin in localized areas, enhancing transcription of certain genes while repressing the transcription of others.	Inframe insertion C>A	
			**Frameshift insertion C>T**	
			Complex mutation G>C	
*TET2*	4q24	It plays a key role in active DNA demethylation. It is also involved in the recruitment of the O-GlcNAc OGT to CpG-rich transcription start sites of active genes, thereby promoting histone H_2_B GlcNAcylation by OGT.	Exon count: 15	Associated with altered sensitivity to bexarotene, Ara-G, tretinoin and VNLG/124.
			**Nonsense substitution A>C**	
			**Missense substitution A>G**	
			Synonymous substitution A>T	
			Inframe deletion C>G	
			**Frameshift deletion G>A**	
			Inframe insertion C>A	
			**Frameshift insertion C>T**	
			Complex mutation G>C	

SETBP1, SET Binding Protein 1; sAML, secondary acute myeloid leukemia; MDS, myelodysplastic syndrome; CMML, chronic myelomonocytic leukemia; pAML, primary acute myeloid leukemia; ETNK1, ethanolamine kinase 1; CSF3R, colony stimulating factor 3 receptor; SRSF2, serine and arginine rich splicing factor 2; NRAS, neuroblastoma RAS viral oncogene; ASXL1, Additional Sex Combs Like 1; TET2, Ten-eleven Translocation 2; OGT, O-linked N-acetylglucosamine transferase; Ara-G, 9-β-D- arabinofuranosylguanine, VNLG/124, 4-(butanoyloxymethyl)phenyl(2 E,4 E,6 E,8 E)-3,7-dimethyl-9-(2,6,6-trimethylcyclohex-1-enyl)nona-2,4,6,8-tetraenoate

Frequently encountered mutations are reported in bold.

**Table 3:** Actionable and non-actionable mutations in aCML.

Mutated gene	Actionable	Potential targeted therapy	Comments
*SETBP1*	No	NA	No reports on the development of agents targeting SETBP1.
*ETNK1*	Possibly	P-Et	It has been shown in ETNK1 overexpression models and patient samples that P-Et is able to fully counteract the metabolic effects of ETNK1 overexpression (24).
*CSF3R*	Yes	Ruxoitinib for *CSF3R* membrane proximal mutation	After a few case reports on the successful use of ruxolitinib in patients with CSF3R mutations, a phase II study including 23 patients with aCML, six of whom carried an activating mutation of CSF3R reported an overall response rate of only 8.7% (18).
		Dasatinib for *CSF3R* truncating mutation	*In vitro* studies with dasatinib on cell lines with truncating mutations of *CSF3R* have shown sensitivity of the cells to the drug (25, 26), but no *in vivo* reports have supported these findings.
*SRSF2*	Possibly	CTX-712	CTX-712 is a novel Clk Inhibitor that has been found to exert an anti-tumor effect in an SRSF2-mutated xenograft model (26).
*NRAS*	Yes	Trametinib	A phase I/II nonrandomized study has shown clinical activity of trametinib in several RAS-mutated myeloid malignancies (27).
*ASXL1*	Possibly	BAP1 catalytic inhibitors	BAP1 catalytic inhibitors have been shown to inhibit truncated-ASXL1-driven leukemic gene expression and halt tumor progression *in vivo* (28).
*TET2*	Possibly	TET-selective small molecule inhibitor	Treatment with a TET-selective small molecule inhibitor has been found to suppress the clonal evolution of TET2 mutant cells in murine models and human leukemia xenografts (29).

aCML, atypical chronic myelogenous leukemia; BCR/ABL1 negative; P-Et, phosphoethanolamine SETBP1, SET Binding Protein 1; NA, not applicable; ETNK1, ethanolamine kinase 1; CSF3R, colony stimulating factor 3 receptor; SRSF2, serine and arginine rich splicing factor 2; NRAS, neuroblastoma RAS viral oncogene; ASXL1, Additional Sex Combs Like 1; BAP1, BRCA1 associated protein 1; TET2, Ten-eleven translocation.

### SET Binding Protein 1 (SETBP1)

The most frequently identified mutated gene in aCML, in up to one third of cases, is *SETBP1* (15, 20, 21, 30). SETBP1 binds to SET which inhibits the tumor suppressor PP2A. This binding protects SET from cleavage, thus repressing PP2A activity. Mutations of *SETBP1* lead to ubiquitination and, thus, degradation of the protein, increasing SET expression, and, consequently, cellular proliferation through inhibition of PP2A. *SETPB1* mutations have been correlated with more pronounced dysplasia, higher WBC counts, more severe anemia and thrombocytopenia, as well as a worse prognosis (20, 21). Moreover, *SETBP1* mutations have been associated with *ASXL1* and *CBL* mutations while they have been found to be mutually exclusive with *JAK2* and *TET2* mutations (21). *SETBP1* mutations have been incorporated as a supportive criterion for aCML in the WHO 2016 diagnostic criteria.

### Ethanolamine Kinase 1 (ETNK1)

ETNK1 is responsible for the phosphorylation of ethanolamine to phosphoethanolamine as part of the Kennedy pathway, which is the main metabolic route for the synthesis of phosphatidylethanolamine and phosphatidylcholine (31). The presence of phosphatidylethanolamine is crucial for cytokinesis and cells lacking phosphatidylethanolamine are unable to complete the mitotic process while reduced intracellular phosphoethanolamine causes hyperactivation of the mitochondria, ROS production, and Histone H2AX phosphorylation, ultimately leading to DNA damage. Recurrent somatic mutations of *ETNK1* have been found in about 13% of patients with aCML (32). A recent study on 43 aCML samples identified *ETNK1* mutations in 16.2% of the analyzed samples while it also suggested that whereas *ETNK1* mutations are an early event in the clonal evolution history of the disease, *SETBP1* mutations are usually a late event (33). Although the presence of *ETNK1* mutations has been considered as a relatively specific finding for aCML, leading to their inclusion in the 2016 WHO classification as a support criterion for the diagnosis of the disease, they have also been found in chronic myelomonocytic leukemia (CMML) and systemic mastocytosis with eosinophilia (34) while they were recently described in diffuse large B-cell lymphoma (35). Nevertheless, the interest in *ETNK1* mutations remains high due to the fact that phosphoethanolamine, the metabolic product of ETNK1, has been shown to negatively control mitochondrial activity, thus restoring a normal phenotype (24).

### Colony Stimulating Factor 3 Receptor (CSF3R)

Although, in an early study, *CSF3R* mutations had been reported in 59% of patients with aCML and CNL (25, 36), further studies did not confirm this high frequency, and it is now believed that *CSF3R*-mutated aCML is in fact rare (19, 37). Nevertheless, these mutations have been proposed to activate either the JAK-STAT pathway (*CSF3R* membrane proximal mutation), or the SRC-kinase signaling pathway (*CSF3R* truncating mutation), thus being targetable with ruxolitinib or dasatinib, respectively (38). Moreover, due to the extremely high frequency of CSF3R mutations in CNL (39), these mutations constitute a criterion for the diagnosis of CNL and, along with other morphological and clinical features, help distinguish CNL from aCML In fact, the identification of a CSF3R mutation in a patient with hepatosplenomegaly, hyperleukocytosis with prominence of neutrophils, low percentage of peripheral blood immature granulocytes and myeloblasts, along with minimal or absent dysgranulopoiesis in the bone marrow strongly favors the diagnosis of CNL over aCML.

### Serine and Arginine Rich Splicing Factor 2 (SRSF2)

*SRSF2* mutations have been reported in 12%, 13.5%, and 40% of aCML cases in three studies (15, 33, 37). In the study by Patnaik et al., the presence of *SRSF2* mutations in three of the examined cases did not affect OS, while one patient had a concomitant *TET2* mutation. On the other hand, in the study by Meggendorfer et al., it was shown that *SRSF2* mutations tended to coexist with *SETBP1* and *ASXL1* mutations.

### *RAS* Mutations

*NRAS* mutations resulting is constitutive activation of the MAPK pathway, thus, promoting malignant cell survival and proliferation, have been detected in up to one third of patients with aCML (19, 40). Moreover, it has been shown that enhanced MAPK signaling is crucial to leukemogenesis by *CSF3R* mutants in CNL (41).

### Other Gene Mutations

Mutations of *ASXL1* and *TET2* are found in over 15% of cases with aCML. In one study, *TET2* mutations were correlated with lower OS along with advanced age and low hemoglobin level. On the other hand, *ASXL1* is considered to be the most commonly mutated gene in aCML (28%), but it has also been shown not to affect overall survival (OS) despite the detrimental effect on survival in CMML, CNL, and PMF (37). Mutations of *ASXL1* have been found to accompany spliceosome gene mutations in as high as 65% of cases (21), a fact supporting the hypothesis of serial accumulation of mutations in the malignant clone, that seems to apply to all MDS/MPNs (42). It should be noted though, that the correlation of OS with the mutational status has been based in small patient series and should, therefore, be considered with caution.

## Treatment Approaches for aCML

As already mentioned, there is no standard of care for the management of patients with aCML. The rarity of the disease has led to its misclassification, resulting in the lack of a universally accepted risk stratification system that would lead to the formulation of recommendations for risk-based treatment strategies. Treatment choices are based on the results of small trials and patient series while some classical therapeutic options have never actually been studied and their use is only based on their efficacy in other MDS/MPN. Recently, the molecular profile of the disease has led to the emergence of new treatment options based on targetable mutations, but their use is mostly based on preclinical data or case reports. Most patients require some kind of treatment since their initial presentation, due to the accelerating leukocytosis and the deteriorating anemia, splenomegaly, and constitutional symptoms, thus a wait-and-watch strategy is rarely advisable.

As already mentioned, there is a lack of risk stratification systems for aCML, mainly due to the rarity of the condition and to the absence of large studies evaluating prognosis. Nevertheless, there have been some efforts to identify adverse prognostic factors and to stratify the patients according to their risk for AML transformation and death. In one of the largest studies in aCML, the authors analyzed the prognostic characteristics of 76 treatment-naïve patients with *BCR/ABL1* negative CML. Multivariate analysis identified age >65 years, hemoglobin level <10 g/dL, and WBC>50x10^9^/L as independent prognostic factors of poor survival (43). In the same study it was shown that treatment did not significantly affect survival. Moreover, a simple scoring system assigning one point to each one of the three above-mentioned independent prognostic factors was designed to stratify patients according to their expected survival. The system stratified the patients into two risk-groups (low risk with 0-1 point and high risk with >1 points), with a corresponding median survival of 38 months *versus* 9 months (p<0.01). Although this risk stratification system has not been widely used, the value of its prognostic parameters has been confirmed in more recent studies. Thus, in a study of 55 patients with aCML (44) multivariate analysis associated shorter survival with age >65 years, female sex, WBC>50x10^9^/L, and presence of circulating immature precursors while the hemoglobin level did not retain its statistical significance. The authors also evaluated the risk of leukemic transformation that was found to be higher in patients with palpable hepatosplenomegaly, monocytosis, bone marrow blasts >5%, marked dyserythropoiesis, and transfusion dependence. Moreover, they tried to validate the prognostic scoring system by Onida et al. and reported that, in their cohort, it could not identify poor survival but identified patients at higher risk for AML transformation. A smaller study reported longer survival rates in patients with normal platelet counts and hemoglobin level >10 g/dL (45) while in a study of 65 patients, WBC>50x10^9^/L, and a high blood immature myeloid cell count and bone marrow blast count (as continuous variables) were correlated with lower OS in univariate analysis. However, no multivariate analysis was carried out (19). Finally, in the most recent study in aCML, age >67 years, hemoglobin level <10 g/dL, and *TET2* mutations were correlated with lower survival rates in multivariate analysis (37). The authors also provided a two-group prognostic model based on the above parameters, with a median OS of 18 and seven months, respectively. **Table 4** summarizes the proposed prognostic factors for aCML by the above referenced studies.

**Table 4:** Factors correlated with lower overall survival in patients with atypical chronic myelogenous leukemia, *BCR/ABL1* negative.

	Onida et al	Breccia et al	Wang et al	Hernandez et al	Patnaik et al
Number of patients, N	76	55	65	11	25
Median OS (months)	24	25	21.4	14	10.8
Age (years)	>65	>65	–	–	>67
Gender	–	F	–	–	NS
Hemoglobin level (g/dL)	<10	NS	–	<10	<10
Dyserythropoiesis	–	present*	–	–	–
Transfusion dependence	–	present*	–	–	NS
WBC (x10^9^/L)	>50	>50	>50	–	NS
Monocyte count	>1.0x10^9^/L	3-8%*		–	–
Platelet count (x10^9^/L)	–	–	NS	<140	NS
Blood immature myeloid cells (%)	>10	present	↑	–	NS
Bone marrow blasts (%)	–	>5*	↑	–	NS
LDH level (U/mL)	>2000	–	NS	–	–
Hepatosplenomegaly	–	Present*	–	–	NS
Gene mutations	–	–	–	–	TET2

*Risk factor for acute myeloid leukemia transformation.

OS, overall survival; F, female; NS, not significant; WBC, white blood cell; LDH, lactated dehydrogenase; ↑, high (as a continuous variable).

In conclusion, most of the studies reporting on prognostic factors in aCML agree that advanced age, anemia, hyperleukocytosis, and presence of immature myeloid cells in the peripheral blood adversely affect OS. It is becoming more and more obvious that the underlying molecular mechanisms may be well correlated with prognosis; thus, further analysis of the molecular footprint of the disease will allow the design of more accurate risk stratification systems that will define the treatment needs of each patient. The available treatment options for patients with aCML are presented in detail in the following paragraphs.

### Allogeneic Hematopoietic Stem Cell Transplantation (Allo-HSCT)

Allo-HSCT should be considered in all eligible patients, given the unfavorable prognosis of aCML. Nevertheless, the age of the patients is usually a prohibitive factor while at the same time there is limited reliable data on the efficacy of allo-HSCT in patients with aCML. Several small studies of retrospective nature have been published. In a study of nine transplanted patients (four from HLA-identical siblings, four from HLA-compatible unrelated donors and one from a twin sibling) followed for a median period of 55 months, all nine achieved complete chimerism and remained in complete remission (CR) while, with the exception of one patient who died from cerebral toxoplasmosis, the remaining eight were alive at the time of the analysis (46). In another study of seven patients with aCML treated with allo-HSCT, after a follow-up period of 17.5 months, only two were alive; one had died due to aCML, and the remaining four due to transplantation related complications (47). In an analysis of 42 cases reported to the European Society for Blood and Marrow Transplantation (EBMT) registry, donors were HLA-identical siblings in 64% and matched unrelated in 36% of the cases. A CR was observed in 87% of patients and the 5-year relapse-free survival was 36%. Younger patients with low EBMT risk scores were found to be the best candidates for allo-HSCT (43). In a recent study of 14 patients with aCML treated with allo-HSCT, 13/14 had received first-line therapy (10/14 with hydroxyurea) with variable responses, 8/13 had received second-line therapy before transplant, and 4/8 third-line therapy while at least two had progressed to acute myelogenous leukemia (AML) before allo-HSCT. Five, seven, and two patients received an allograft from HLA-matched related, unrelated marrow, and unrelated cord blood donors, respectively. A myeloablative regimen was used in 11/14 patients. Among patients with neutrophil engraftment, 9/13 achieved a CR and the 1-year OS was 54.4%, being higher in univariate analysis in patients with related donors, <5% myeloblasts in the peripheral blood, and a Karnofsky performance status of ≤80% (48).

Questions on the donor source, the correct timing (i.e. upfront transplantation *versus* transplantation after initial treatment to reduce the disease burden), the intensity of the conditioning regimen, the impact of previously administered treatments, and the possible impact of the molecular profile of the patients still remain unanswered. Nevertheless, the advanced age of most patients with aCML probably dictates the use of reduced intensity conditioning regimens while the molecular profile of the patients may be useful for the monitoring of minimal residual disease (49) and as a guide for post-transplant maintenance in cases with targetable mutations.

### Hypomethylating Agents (HMAs)

The use of HMAs in patients with aCML should be a case-by-case approach since patients with striking myeloproliferative features are less likely to respond to HMAs. Although data on the efficacy and safety of HMAs in aCML is limited, the rationale for their use is sound, based on their established activity in CMML and other MDS/MPN. Early data on the use of decitabine in seven patients with aCML and a median age of 67 years showed an OS rate of only 13 months and a 14% 2-year survival rate (50). In a study of 76 patients with aCML treated with several regimens (5 patients treated with an HMA), OS was not affected by the treatment choice, but no information is specifically provided for the prognosis of patients treated with HMAs (51). Decitabine has also been administered in four patients with aCML and after a median of 2.5 cycles of treatment and a median follow-up of 13 months, three patients were still alive (one of them eventually treated with allo-HSCT) (52). Finally, in a report of five patients with aCML treated with HMAs, the best achieved response was stable disease (37), while a few case reports give mixed results on the efficacy of HMAs. Thus, a general rule would be that the use of HMAs in patients with aCML should be restricted in elderly patients with prominent dysplastic and more subtle myeloproliferative features, or as a bridge to a transplant.

### Cytoreduction and Intensive Chemotherapy

Historically, hydroxyurea has been the mainstay of treatment for patients with aCML and other MDS/MPNs. In recent reports, it remains the most widely used factor for the management of myeloproliferation in aCML and it can effectively control leukocytosis and splenomegaly, but responses are typically short-lived. In an early study on 11 patients with aCML, nine were treated with hydroxyurea achieving a partial remission (8). In a study of 55 patients with aCML, 48 (87.3%) patients had been treated with hydroxyurea, but the effect of treatment on OS was not further discussed by the authors (44). Furthermore, in a study on the characteristics and outcome of 76 patients with aCML, the authors stated that 53% of the patients had already been treated with hydroxyurea or busulfan at the time of their referral to the study center while nine of them were also treated with hydroxyurea after their referral. Although there was no specific mention of the outcome per regimen, the authors reported that there was no significant difference in OS between treated and untreated patients (43). Finally, in a more recent study of 25 patients, hydroxyurea had been administered in 15 (60.0%) patients, but treatment outcomes were not evaluated (37). Busulfan and low-dose cytarabine have also been used in the treatment of patients with aCM while intensive chemotherapy is not a standard treatment option for patients with aCML and should be reserved for patients with leukemic transformation.

### Ruxolitinib

Activating mutations in *CSF3R* have been found to drive myeloproliferative disorders resembling aCML and CNL in mouse models, where these mutations are sensitive to JAK inhibition that may effectively reduce the WBC count and the spleen size (53). Preliminary data has shown that treatment of aCML/CNL cells carrying activating mutations of *CSF3R* with ruxolitinib resulted in inhibition of cell growth while a patient with CNL bearing the *CSF3R* T618I mutation was effectively treated with ruxolitinib (25). Although the experience with ruxolitinib was limited to case reports, a recent phase II study was conducted to investigate the hematologic response to ruxolitinib in patients with aCML/CNL. The study included 23 patients with aCML, six of whom carried an activating mutation of *CSF3R* (T618I, T640N, or T615A). Response (PR and CR) was reported in only two (8.7%) patients while no serious adverse events attributed to ruxolitinib were observed (54). The results of this trial reduced the initial enthusiasm on the efficacy of targeting *CSF3R* mutations with ruxolotinib in aCML, especially since these mutations proved to be rather rare in this condition. The use of ruxolitinib as a bridge to transplant, though, is still a valid choice in an effort to reduce the WBC count and splenomegaly following the paradigm of myelofibrosis although clinical trials are still lacking.

### Dasatinib

*CSF3R* truncation mutations have been shown to activate the SRC family pathway offering an option of targeted therapy with dasatinib. This SRC family kinase inhibitor used in CML has been proposed to potentially have therapeutic value in aCML with *CSF3R* truncation mutations since *in vitro* studies of cell lines with such mutations have shown sensitivity of the cells to the drug (18, 25). However, these preclinical data have not been supported by *in vivo* reports to date.

### Trametinib

The mitogen-activated protein kinase kinase (MEK) inhibitor trametinib, approved for the treatment of advanced or metastatic melanoma, has been proven active against *NRAS*-mutated AML in human cell lines and murine models (55). Moreover, it has been shown that MEK inhibition with trametinib is sufficient to suppress CNL induced by *CSF3R* mutations, highlighting a MAPK-dependent mechanism of CSF3R-induced pathogenesis that is valid at least in CNL (41). A case report of a patient with aCML responding to trametinib (56) and a phase I/II nonrandomized study showing clinical activity of trametinib in several RAS-mutated myeloid malignancies (27) have confirmed these preclinical data although the beneficial effect of the drug in reducing the blast count was not translated into a survival benefit. Further studies with trametinib as monotherapy or in combination with other targeted therapies are needed to test its efficacy in aCML.

### Other Agents

Interferon has been used in aCML with mixed results. In an early study of 14 patients, interferon-α was administered to seven patients following treatment with hydroxyurea, and CR was achieved in five (57). In another study on the characteristics and outcome of patients with aCML, interferon-α and interferon-γ were used as single-agent therapy in 17 patients with aCML; nevertheless, response rates were not presented (43). Moreover, among 11 patients with aCML, six were treated with interferon-α, two of whom achieved a CR lasting for 9 and 40 months (8). In another early study, out of 14 patients treated with interferon-α, six (43%) of them responded (five with a CR) with a duration of response ranging from 1 to >100 months (58). Finally, among five patients with aCML treated with peg-interferon-α-2b in the context of a phase II study, two responded to treatment achieving a CR after three months of treatment (59). The above-referenced results show that the use of interferon-α and its pegylated formulations should be further investigated.

### Supportive and Palliative Treatment

Supportive treatment should be offered to all patients in the form of erythropoiesis-stimulating agents (ESAs) and red blood cell transfusions. The use of ESAs in terms of efficacy and safety has never been specifically studied for aCML. Thus, factors predicting response to ESAs have not been established. Moreover, heavily transfused patients should probably also receive iron chelation therapy, depending on their estimated prognosis, but this strategy is not supported by any published data. Finally, the use of corticosteroids, danazol, and thalidomide or lenalidomide to treat anemia has never been studied or reported in patients of aCML. Splenectomy or splenic irradiation have been scarcely used in the past as palliative measures in patients with aCML, with no disease improvement (8, 50, 60). Thus, they should be avoided since any possible benefit is cancelled out by potentially severe complications, such as bleeding, thrombosis, infection, and potential acceleration of leukocytosis and/or hepatomegaly (22).

### Conclusions on Treatment Approach

Based on the available data, the general principals of the management of patients with aCML can be summarized into the following.

- Allo-HSCT should be offered to all eligible patients.
- Myeloid mutation panel testing should be performed to all patients to detect potentially targetable mutations.
- Although clinical trials focusing on patients with aCML are rare, inclusion in a clinical trial should be an early choice, especially for patients without targetable mutations.
- In patients not eligible for allo-HSCT, the treatment should focus on addressing the patient’s major clinical issues (constitutional symptoms, anemia, organomegaly) and potentially on decreasing the possibility of progression to AML although supporting data about the latter is lacking.
- Treatment strategies applicable to patients with MDS or MPN can be selected on a case-by-case approach.
- Given the poor prognosis of aCML, initiation of treatment is generally favored over a watch-and-wait strategy.

The prognosis of patients with aCML remains poor and cases with long survival are the exception to the rule. Further identification of the molecular footprint of the disease may allow for the emergence of new targeted treatment choices that may reduce the risk for AML transformation and prolong survival.

## Author Contributions

PD has drafted the manuscript and N-AV has critically revised the manuscript. All authors contributed to the article and approved the submitted version.

## Conflict of Interest

The authors declare that the research was conducted in the absence of any commercial or financial relationships that could be construed as a potential conflict of interest.

## Publisher’s Note

All claims expressed in this article are solely those of the authors and do not necessarily represent those of their affiliated organizations, or those of the publisher, the editors and the reviewers. Any product that may be evaluated in this article, or claim that may be made by its manufacturer, is not guaranteed or endorsed by the publisher.
